# Breast cancer screening adhesion among migrants: a matter of communication strategy?

**DOI:** 10.1093/eurpub/ckac130.174

**Published:** 2022-10-25

**Authors:** S Banzola, A Corsaro, S Capitani, S Vesco, L Dodi, O Serra, N Piazza, S Onesti, MA Salvati, A Musolino

**Affiliations:** 1 Migrant Health Unit, Spazio Salute Immigrati, Local Health Unit, Parma, Italy; 2 Public Health Department, Local Health Unit, Parma, Italy; 3 Press and Communication Office, University Hospital, Parma, Italy; 4 Social Health Unit, Center for Immigration Asylum and Cooperation, CIAC Onlus, Parma, Italy; 5 Healthcare Management Direction, Local Health Unit, Parma, Italy; 6 Breast Unit, University Hospital and Local Health Unit, Parma, Italy; 7 Cervical Screening Center, UOC Salute Donna, Local Health Unit, Parma, Italy

## Abstract

**Background:**

Migrants’ engagement to cancer screening programs is a relevant issue for universalistic health systems. To increase breast cancer screening coverage among migrant women, a public-private partnership involving a multidisciplinary team of Primary Care, Public Health, Hospital and private social workers has been built up in a district in Italy. The team worked in two steps, planning health promotion (HP) meetings addressing women in refugees’ reception programs and a web-based workshop involving intercultural mediators (IMs) and community health promoters.

**Objectives:**

The workshop, involving 10 professionals among IMs and community health promoters, realized in 3 online meetings during March ‘22, aimed at identifying communication tools to enable migrants’ participation to breast cancer screening and increasing health literacy (HL) and cultural competence (CC) among the team. A participatory approach, supported by learning methods, such as storytelling and role-play, has been adopted to identify the major barriers to access to screening and public health messages. Participants worked on critical words and concepts, highlighted during HP meetings, accounting for HL, literacy, language skills, communication techniques and different perspectives about health and prevention.

**Results:**

Several barriers, such as lack of knowledge on preventive initiatives and different approaches to health, decrease the perception of cancer risk. Others, like family and work duties, influence the adhesion. Fear or shame about the exam and linguistic issues are further hampering factors. Participants pointed out text, audio and video messages, in Italian and native plain language, as useful tools to explain the screening procedure and give relevant and practical information supported by simple and clear illustrations to diffuse via WhatsApp.

**Conclusions:**

The intervention enabled the team to improve HL and CC defining suitable communication strategies for cancer screening programs.

**Key messages:**

- Within the team building process, professionals, from health and private sectors, improved communication skills and awareness of the role played by HL and CC in reducing health disparities.
- Active learning methods, such as role-play and storytelling, resulted to be strategic for the participatory approach and to achieve the objectives.

